# Climate change: what competencies and which medical education and training approaches?

**DOI:** 10.1186/1472-6920-10-31

**Published:** 2010-04-30

**Authors:** Erica J Bell

**Affiliations:** 1 University Department of Rural Health, University of Tasmania, 18 Elizabeth Street, Hobart, 7000, Australia

## Abstract

**Background:**

Much research has been devoted to identifying healthcare needs in a climate-changing world. However, while there are now global and national policy statements about the importance of health workforce development for climate change, little has been published about what competencies might be demanded of practitioners in a climate-changing world. In such a context, this debate and discussion paper aims to explore the nature of key competencies and related opportunities for teaching climate change in medical education and training. Particular emphasis is made on preparation for practice in rural and remote regions likely to be greatly affected by climate change.

**Discussion:**

The paper describes what kinds of competencies for climate change might be included in medical education and training. It explores which curricula, teaching, learning and assessment approaches might be involved. Rather than arguing for major changes to medical education and training, this paper explores well established precedents to offer practical suggestions for where a particular kind of literacy--eco-medical literacy--and related competencies could be naturally integrated into existing elements of medical education and training.

**Summary:**

The health effects of climate change have, generally, not yet been integrated into medical education and training systems. However, the necessary competencies could be taught by building on existing models, best practice and innovative traditions in medicine. Even in crowded curricula, climate change offers an opportunity to reinforce and extend understandings of how interactions between people and place affect health.

## Background

Health workforce development for climate change has become a pressing matter. It is an emerging priority in international policy guidelines for health workforce development produced by the Global Health Workforce Alliance [1] and has featured in recent international policy forums such as the 2008 Geneva Health Forum. The work of the World Health Organisation now includes a focus on the ways in which global health issues such as climate change can be included in health workforce education programs for developing countries. Developed countries are also beginning to include health workforce development for climate change in their national policy statements. For example, education and training of the health workforce features in the UK Department of Health guidance document *The health impact of climate change: promoting sustainable communities *[2]. Health workforce development is identified as a priority area in the USA's sentinel policy statement, the *CDC Policy on Climate Change and Public Health *[3]. Australia's premier policy document *Human Health and Climate Change--National Adaptation Research Plan *takes a strong focus on workforce education training and development [4].

Health professional advocacy groups such as The British Medical Association have supported the Commission of the European Communities *Green paper: On the European Workforce for Health *[5] which asserts that climate change is a major factor shaping public health capacity. The Commission stated that European Union member states will have to assess the kinds of specialist skills that will be needed in the future in response to shifting patterns of disease caused by climate change [6]. Public health organisations, such as the USA's Association of Schools of Public Health (ASPH), which represents the forty-one Council on Education for Public Health (CEPH) accredited schools of public health in north America, are identifying preparedness for climate change as a key challenge of their definitions of 21^st ^Century health workforce crises [7]. This is so in a context where a recent nationwide survey of senior public health officials in the USA found that the majority of them had already observed the effects of climate change but felt they and their agencies lacked the expertise to develop adaptation strategies and plans [8].

Practice is often ahead of teaching and research: it is likely that, in rural and remote regions now being affected by climate change, doctors are already acquiring understandings of interactions between climate change and health they were not taught at medical school [9,10]. This extends a challenge to educators of health professionals to question what kinds of new competencies might be demanded by a climate-changing world, particularly for communities that will be most affected by climate change. However, in a crowded curriculum, what are the opportunities for teaching such 'eco-medical' competencies in a way that builds on existing approaches to medical education and training?

Rather than arguing that medical education needs to make major changes to an already crowded curriculum, this debate paper explores where this eco-medical literacy might be integrated into existing curriculum, teaching, learning and assessment. This approach is consistent with the October 2009 comprehensive statement adopted by the World Medical Association--the *Declaration of Delhi on Health and Climate Change*--which emphasises extending environmental health/medicine and public health to meet the needs of all students in health-related disciplines [11].

## Discussion

### The knowns and unknowns of climate change

It took a long time for the health effects of climate change to be recognised. These health effects are documented by the Intergovernmental Panel on Climate Change; more recently *The Lancet *has published a watershed paper on climate change supporting the view that it represents the biggest potential threat to human health in the 21^st ^Century, with 5.5 million DALYs (disability adjusted life years--a measure of human health) lost in one year of this century alone [12].

Climate change is bringing health effects from extreme weather events such as heatwaves, cyclones and drought: injuries and mortality, as well as food supply problems, particularly for people in rural and remote regions. There are also likely to be increases in a whole host of health conditions indirectly linked to global warming. These include cholera and other waterborne diseases linked to water supply contamination; insect-borne diseases such as tick-borne encephalitis; asthma and other conditions linked to air quality and unseasonal influences on weeds and pollens [13-15].

Much less is known about how regional conditions will shape the nature and trajectory of these health phenomena [14]. Regional variation in such direct and indirect health effects is, however, very likely. The prospect of even greater inequalities in health outcomes for many rural and remote communities is also looming [16,17]. The role of environment in shaping health will become more complex and dynamic as eco-systems become dysfunctional and unpredictable [18]. The incidence, range and seasonality of many existing conditions will be altered and new strains of virus and microbes may appear [10,17].

Research on what medical education and training should do about climate change is very limited. Of the over 800 published papers on climate change and health, mostly by the developed world, only a handful offer general discussion of the implications of climate change for medical education and training [10,18,19].

### Defining eco-medical literacy

The importance of different kinds of literacies is well known to medicine. The World Health Organisation (WHO) has defined health literacy as a patient's ability to gain access to, understand, and use information to improve health [19]. Medical education and training seeks to give doctors different kinds of literacies, both clinical and non clinical. For example, doctors must develop scientific literacy, as well as technological and cultural literacies, important to healthcare. The climate change literature suggests these literacies will not be enough in the future, that another kind of environmental literacy will be needed.

This environmental literacy can be understood as an extension of the disciplines of occupational and environmental medicine, represented in a growing body of scholarly literature, as well as courses and textbooks [20] and organisations such as the American College of Occupational and Environmental Medicine. Environmental medicine for clinical practice has been defined as a style of medical detective work about the environment, particularly toxins, to improve medical treatment, particularly for chronic conditions such as asthma and allergies [21]. Thus environmental medicine courses offer a key opportunity for integrating climate change into health professionals' courses.

The broad church of environmental medicine also includes the growing subdiscipline of 'ecosystem health', focussing on the ways in which health is shaped by 'the capacity for ecosystems to be self-sustaining and to carry out all of their normal functions'.(353) [22] This conceptualisation of environmental medicine is associated with approaches in some medical education schools, for example, the eco-health program at the Faculty of Medicine and Dentistry, University of Western Ontario, as well as the work of the International Society for Ecosystem Health [23]. Thus, another opportunity for developing environmental literacy for climate change might be in ecosystem health courses that typically take a wider sustainability focus (of which climate change and its health effects are only a part).

Environmental literacy for climate change can also be defined as a natural extension of rural and remote medical education and training. This is so despite the fact that national curriculum statements of rural and remote practice, as well as many local curriculum documents, currently make no ostensible reference to climate change, even in well-resourced countries such as Australia [24], Canada [25] and the UK [26], which have produced research on climate change as well as rural and remote health [10]. However, national curriculum statements for rural and remote medicine do strongly emphasise relationships between place, community and culture. Medical practice in these communities is seen as distinctive, requiring a specialised set of knowledge and skills involving highly contextualised knowledge, including of relationships between people and country [27,28]. For example, remote medicine for indigenous populations has been defined as

...an emerging discipline with distinct sociological, historical and practice characteristics. Its practice in Australia is characterised by geographical, professional and, often, social isolation of practitioners; a strong multidisciplinary approach; overlapping and changing roles of team members; a relatively high degree of GP substitution; and practitioners requiring public health, emergency and extended clinical skills. These skills and remote health systems need to be suited to working in a cross-cultural context; serving small, dispersed and often highly mobile populations; serving populations with relatively high health needs; and a physical environment of climatic extremes [28].

As such, rural and remote medical education offers another opportunity for integration of climate change into medical education and training.

The 'eco-medical literacy' that relates to climate change can be defined as

...the ability to access, understand, integrate and use information about the health-related ecological effects of climate change to deliver and improve medical services.

Beyond the disciplines of environmental medicine and rural and remote medicine, there are many other signs that such ecological understandings of human health can no longer be considered peripheral to mainstream medical education and training. For example, three important science panels of the Institute of Medicine as well as the National Research Council in the USA have called for greater interdisciplinary integration involving the behavioural and biomedical sciences, towards ecological frameworks for health research and health professional education [29-31]. Many contemporary studies of health professional education point to the limitations of approaches that take a narrow clinical or basic science approach [32]. Thus, environmental literacy and specifically eco-medical literacy for climate change can be considered an important part of producing the well rounded medical professional with extended clinical reasoning and diagnostic skills.

In a context in which the curriculum is already crowded in both undergraduate and postgraduate education, the emphasis should be on using climate change to help meet the aims of existing courses. Climate change can be incorporated into environmental health or public health courses in undergraduate medical education, with brief optional courses in residency and continuing medical education for established practitioners. Postgraduate training should target those who may be considering or already are practicing in regions most affected by climate change.

### Competencies for eco-medical literacy

What kinds of competencies might be needed in a climate-changing world?

The previous discussion on the 'knowns and unknowns' of climate change and health suggests that clinical practice will have to develop in particular ways in response to climate change, particularly in climate change 'hotspots'. For example, details about local eco-system changes may well need to be captured alongside information about presenting conditions in clinical histories. Diagnostic reasoning may be improved by information about how dysfunctional eco-systems operate to shape disease. The puzzle that forms diagnostic reasoning--the information about the aetiology, pathogenesis and epidemiology of health conditions--may need to include information about changing local ecologies in global meltdown. Management plans, treatment protocols and guidelines may need to include the realities of how people are living with climate change. New health infrastructure conditions developed in response to climate change, or the failure of existing infrastructure, may redefine how doctors should case manage with other health, allied health and social services.

Emergency care is also likely to be reshaped by the pressures of climate change. As emergency infrastructure adapts to climate change events, emergency procedures and protocols will alter. Methods for implementing disaster plans, as well as retrospective analysis and development, may need to change rapidly in the light of learning about climate change. This will change what doctors need to know and do. Recent events such as Hurricane Katrina in the USA and the Victorian bushfires in Australia have challenged public health understandings of appropriate responses to extreme weather events. For example, the submission of the Victorian Healthcare Association to the Victorian Bushfires Royal Commission concluded that 'a volatile climate has, and will continue to have, a significant effect on the frequency, severity and gravity of environmental emergencies into the future. Whilst this is difficult to quantify, or predict, it is evident that emergency planning and strategies need to be reviewed and adapted'. It recommended expansion and modification of existing training approaches to better equip health agencies to respond to such climate-influenced emergencies, observing that 'In the future, Black Saturday bushfires may be a more useful case study around which to model training packages' [33].

General medical practice often includes as a substantial component an understanding of community-based factors that shape health and the delivery of health services. In a climate-changing world, general practitioners may also need to adapt and develop their workplaces to help put in place systems for preventing and detecting health conditions. For example, they may need to build specialised skills in detecting and intervening in outbreaks of such conditions as Ross River Virus combining regional surveillance data on climate changes and mosquitoes [34]. General practitioners, particularly in climate change 'hotspots', may need to be able to help develop new regional health reporting methods that account for local environmental variables. They may need to learn new ways of collaborating with public health services and community agencies to help design and implement regionally-responsive population health interventions.

Cultural literacy relevant to delivering quality medical care to indigenous peoples is a critical aspect of the distinctive requirements of remote medical practice [28]. In a climate-changing world, the special relationships that indigenous peoples have with place will shape the effects of climate change on their health [9]. Their methods of adaptation to climate change are likely to be different, socially, economically and politically. The disease profile of indigenous peoples is likely to alter as a function of their response to climate change. This will change the clinical and non clinical competencies that will be useful to delivering medical services to indigenous peoples.

Medical education and training also frequently seeks to impart professional, legal and ethical competencies. In a climate-changing world, such competencies may take a different form. For example, doctors may need to access and share different kinds of information for their professional development. They may need to engage in collaborative learning with those who work in areas beyond health, such as new kinds of environmental specialists performing regional weather forecasting and environmental trouble-shooting. Regional variation in health effects may bring greater responsibilities to share this locally-acquired eco-medical literacy with their immediate peers.

The way in which general practices are conceptualised and run as businesses, and their relationship to their communities, may be different in a climate-changing world. Doctors may need competencies in running eco-friendly businesses that are underpinned by different values for a new age of consequences. For rural and remote practitioners, the increasing divide between the haves and the have nots is likely to bring about ever greater pressures, not just on the delivery of clinical care. There may also be greater pressures on non clinical ethical decision-making about how much and what a doctor should give, to whom, when.

### Developing eco-medical literacy: curriculum

The competencies just described use the climate change literature to extend some of the domains and definitions of competencies given in the Primary Curriculum document of the Australian College of Rural and Remote Medicine (ACRRM) [24]. Internationally speaking, the ACRRM primary curriculum document is the most detailed curriculum model of rural and remote medicine available, nationally accredited for use in general practice education and training. Like other accounts of rural and remote medicine, [27] it emphasises relationships between geography, people, culture, history and medicine in contextualised problem-solving. As such, it offers a well established point-of-departure for developing workable competencies for a climate-changing world.

The following offers an overview of the different domains of rural and remote practice where such competencies can be developed, adapted from the Australian College of Rural and Remote Practice Primary Curriculum document [35]:

#### Domain 1--Core Clinical Knowledge and Skills

Climate change will interact with the already poorer health status of rural and remote communities, influenced by lack of access to health and other services, socio economic factors, as well as cultural factors. This is likely to make new demands on the practitioner's clinical and non clinical skills. For example, primary and secondary clinical care may be impacted as particular vulnerable groups such as socio-economically disadvantaged elderly patients are more affected by climatic extremes such as heatwaves or extended sub-zero temperatures. This may require highly developed communication, diagnostic, clinical procedural and management skills from the practitioner. For example, practitioners may need to consider uncommon diagnoses for new health conditions in regions formerly unaffected by them.

#### Domain 2--Extended Clinical Practice

Rural and remote medicine is already constituted as a multidisciplinary speciality that incorporates knowledge and skills from diverse disciplines as part of a focus on interactions between health, community and place. This domain includes a focus on knowledge about climate change from across the disciplines to respond to, for example, diagnosis of more complex climate-related conditions such as mental health conditions arising from population displacement brought about by extended drought.

#### Domain 3--Emergency Care

Climate change will bring new pressures on the emergency care skills required by health professionals to work in rural and remote communities. Climate-produced disasters such as catastrophic bushfires will require rural and remote practitioners to work with a wide range of emergency personnel (police, fire brigade, ambulance and others) to assess, triage and manage possibly large numbers of patients at difficult-to-access sites. The scale of climate-produced disasters in areas where they have not been experienced before may place additional demands on the rural and remote practitioner's retrieval skills, emergency medical skills, and post-disaster management skills such as grief counselling.

#### Domain 4--Population Health

Climate change is likely to interact with existing population health challenges in rural and remote communities to place demands on practitioners' roles in improving the health of their local communities. Practitioners may be required to have a deeper understanding of the ways in which local ecologies are impacting on the health of their local communities. For example, they may be required to understand how climate change is affecting water and food supplies for local communities. They may also be required to participate in prevention and health promotion linked to adaptation to the effects of climate change as part of, for example, local government heatwave planning.

#### Domain 5--Indigenous Health

Climate change is likely to have particular effects on indigenous populations who already experience worse health outcomes than many other groups. To be effective, health practitioners will need to be able to understand how climate change is shaping the health conditions of indigenous peoples, their health status and their social and emotional well-being. Effective responses will also need to take into account, and be respectful of, the capacities of indigenous peoples to adapt to changing climatic conditions through their own community structures and cultural knowledge.

#### Domain 6--Professional, Legal and Ethical Practice

Climate change will shape the infrastructure, as well as legal and ethical frameworks in which health practitioners work in rural and remote practice. For example, it may bring new legislative requirements in terms of disease notifications as part of early warning systems or new occupational health and safety requirements for flood prone areas. Health practitioners will need to engage in continuous learning and professional development appropriate to a climate-changing world. Practitioners may need to initiate collaborative learning opportunities with other health and environmental professionals to help their local governments respond to events such as extended drought.

#### Domain 7--Rural and Remote Context

Climate change will bring a new focus on the importance of context in rural and remote practice. While context has always been important to medical practice, climate change is likely to place new demands on the practitioner's understanding of the environmental determinants of health. For example, medical professionals may need to develop better knowledge of the underlying mechanisms at work in climate-sensitive health conditions, including health conditions not formerly known to be climate sensitive. This will place greater imposts on the practitioner's resourcefulness and continuous self-improvement in isolated conditions.

The following illustrates the detail of what an eco-medical competency might look like using one of these domains from the ACRRM primary curriculum document as a point of departure: 'Professional, legal and ethical practice'[35]:

#### Domain

Professional, legal and ethical practice

#### Key competency

Engage in continuous learning and professional development appropriate to a climate-changing world.

#### Descriptor

##### Undergraduate education

• Demonstrates knowledge of the relevant professional, legal and ethical obligations to develop and share information about climate change effects on health.

• Demonstrates knowledge of how to access local, national and international information about climate change effects on health, relevant to adapting health services

• Shows how to use information about climate change effects on health to improve decisions about health services delivery

##### Postgraduate training

• Demonstrates knowledge of how to access local, national and international information about climate change effects on health, relevant to adapting health services

• Shows how to use information about climate change effects on health to improve decisions about health services delivery

• Initiates and participates in collaborative learning opportunities with health and environmental professionals active in climate change management. Demonstrates application of this knowledge to adapt and improve health services delivery.

The vast literature on environmental medicine also offers a point of departure for developing curriculum for eco-medical literacy, including in developing countries. Accounts of the clinical practice of environmental medicine suggest competencies in, for example, the detection and management of environmental sensitivities and allergies relevant to a wide range of presenting conditions [21].

The following offers some website links useful to developing competencies for climate change:

The Australian College of Rural and Remote Medicine Primary Curriculum document:

http://www.acrrm.org.au/main.asp?NodeID=90:

The Global Environmental Change and Human Health Project

http://www.essp.org

The American College of Occupational and Environmental Medicine:

http://www.acoem.org

The American Academy of Environmental Medicine:

http://www.aaemonline.org

Despite these precedents, it is clear that a major international study mapping eco-medical literacy is needed. The locally specific, as well as the generic, components of eco-medical literacy can be identified by adapting the standard techniques for identifying medical competencies. For example, analysis of the climate change literature can be supplemented with studies of how practitioners in rural and remote regions are developing healthcare for climate change, such as mental health for farming families experiencing extreme drought ('job analysis') [36,37]. Definitions of the competencies for eco-medical literacy can be refined using consensus-making techniques with practitioners such as the Delphi technique, which has been used to develop competencies for health professions [38].

The foregoing discussion of competencies for eco-health literacy provides some support for the view, expressed in the eco-health medical education literature more generally, that 'ecological concepts, principles and models can be used to facilitate learning of basic biomedical sciences, and to provide an additional analytical dimension useful in diagnosis and treatment'.(p.37) [32] Thus, 'eco-medical' competencies can be used to enhance and extend, rather than replace, elements of existing medical education curriculum, especially for rural and remote practice. Yet their development is likely to raise deeper questions for medical education and training. For example, it is known that nationally accredited curriculum statements must accommodate the distinctiveness of local contexts. In a climate-changing world seeing greater regional diversity, it is likely to be even more important to ensure curriculum development gets the balance right between comparability of standards and local flexibility [10]. In developing countries in particular, any such efforts of curriculum development will need to involve an investment in upskilling medical educators. The evidence of many local curriculum documents, particularly in developing countries, suggests that medical educators often struggle to find practical resources and tools to help them with the basic tasks of curriculum design, let alone developing definitions of eco-medical literacy.

### Teaching, learning and assessment of eco-medical literacy

Best practice approaches emphasising interactions between medicine and environmental science and other disciplines could be enhanced by the teaching and learning of eco-medical literacy. These could take a strong focus on learning in context, using established methods such as interactive learning modules, *in situ *clinical skills learning, problem-based learning, case scenarios, journals, and so on. For example, reflective practices for a climate-changing world could be encouraged by the use of journals where students in rural and remote training sites record their observations of environmental factors shaping health issues.

Assessment of eco-medical literacy could also occur via the well-known assessment techniques, from portfolios to structured examinations. Eco-medical literacy would most likely be demonstrated in assessment contexts that measure performance in complex, multidisciplinary, higher order problem-solving. Ideally, this would involve integrated assessment of global performance using criteria-based assessment guidelines, rather than numerical grades or 'yes/no' checklists of competencies that have known problems in assessing more complex competencies [39]. Thus, the teaching of eco-medical literacy could be used to reinforce higher order problem-solving competencies using criteria-based assessment practices.

Some medical education schools have already established courses that offer a point of departure for developing education techniques for a climate-changing world. The 'eco-system health' program at the University of Western Ontario offers one of the more well-established models for teaching, learning and assessment of eco-medical literacy. It was introduced at that university in the late 1990s, integrating multidisciplinary environmental knowledge into traditional undergraduate 'mainstream' medical education. In this program medical educators have developed innovative approaches to the teaching of, for example, food and water-borne diseases and related issues of food security, the management of natural disasters and human health, and other climate-change issues. Learning experiences include student reflection on complex, varied information about the environment in ways that encourage them to make intuitive links between that information and health conditions--for example, health case studies of disadvantaged peoples displaced by hurricanes and related information about geography, social, political and economic conditions in the region [22]. Other case-based ecological approaches using groups at the University of Western Ontario have included consideration of, for example, regional effects of *E. coli 0157.H7 *as a waterborne disease, as part of an undergraduate ecosystem health elective [40]. Thus, eco-medical literacy could offer an opportunity to reinforce and extend well-established contextualised assessment and learning approaches, especially base-based scenarios.

As Green and colleagues have observed, the best approach to integrating climate change into courses will depend on a range of factors, including whether teaching about environmental determinants of health is already in the curricula. Educators can introduce specific units or they can build climate change considerations into the existing curricula by extension [41]. Thus, integrating climate change into courses for healthcare professionals need not involve a major change to health workforce education and training provided there are foundational ecological emphasises already present. Medical schools such as the John A Burns School of Medicine at the University of Hawaii, which is building a strong Division of Ecology and Health, consistent with its vision of community-based service to the Asia-Pacific region, are likely to be well positioned to more quickly integrate emerging climate change issues than schools that do not include a well-developed eco-health approach [32].

## Summary

Much of the work that needs to be done extending the eco-medical literacy of doctors is about building on existing models, best practice and traditions in medicine. The health effects of climate change have, generally, not yet been integrated into medical education and training systems. This debate paper has aimed to identify key opportunities for integrating climate change into curriculum, teaching, learning and assessment. Rather than providing the detail of 'how to' teach climate change, it has drawn attention to where the main opportunities lie for developing climate change in medical education and training. The empirical validation of teaching approaches for a climate-changing world lies in the future. Yet, even in crowded curricula, climate change offers an opportunity to reinforce and extend understandings of how interactions between people and place affect health.

## Competing interests

The authors declare that they have no competing interests.

## Author information

Dr Erica Bell is A/Co-Director and Deputy Director at the University Department of Rural Health at the University of Tasmania. She has produced 40 academic journal papers and conference presentations/papers across subspecialities of health, as well as 44 applied research reports that developed policy and practices across health and education. Her book *Research for Health Policy *was published by Oxford University Press in 2009; she is currently editor of a volume on rural child health and climate change.

## Pre-publication history

The pre-publication history for this paper can be accessed here:

http://www.biomedcentral.com/1472-6920/10/31/prepub
